# Variants of ERCC5 and the outcome of platinum-based regimens in non-small cell lung cancer: a prospective cohort study

**DOI:** 10.1007/s12032-022-01741-9

**Published:** 2022-07-19

**Authors:** Esraa S. Abdalkhalek, Lamia M. El Wakeel, Ahmed A. Nagy, Nagwa A. Sabri

**Affiliations:** 1 Clinical Pharmacy Department, Faculty of Pharmacy, Ain Shams University, African Union Organization Street, Abbaseya, Cairo, Egypt; 2 Department of Clinical Oncology, Faculty of Medicine, Ain Shams University, Abbaseya, Cairo, Egypt

**Keywords:** ERCC5, Non-small cell lung cancer, Platinum, rs751402, rs1047768

## Abstract

Excision repair complementary complex 5 (ERCC5) is an important component in the repair pathway of platinum-induced damage. The current study evaluated the effect of ERCC5 variants (rs751402 and rs1047768) on the clinical outcome of platinum-based regimens in non-small cell lung cancer (NSCLC) patients. A prospective, cohort study was conducted on 57 newly diagnosed NSCLC Egyptian patients. Patients received either cisplatin or carboplatin-based chemotherapy. DNA was extracted and the variants were analyzed using real time PCR. This study found no significant difference between the studied variants and patients’ response to chemotherapy, progression-free survival (PFS) or overall survival (OS). However, a statistically significant association was found between the histologic subtypes and the studied variants (*p* = 0.028 and 0.018 for rs751402 and rs1047768, respectively). A statistically significant association was evident between the type of the allele present in the studied polymorphisms, *p* value = 0.000040. Moreover, the minor allele frequency (MAF) of the studied variants rs751402 and rs1047768 were similar to those of African and European populations, respectively. Results of this study have concluded that ERCC5 variants did not affect the clinical outcome of platinum-based chemotherapy in NSCLC. A significant coinheritance was found between the two variants of ERCC5. Moreover, the similarity between the MAF of the studied variants and the African or European population can guide future research when extrapolating data from African European populations to their Egyptian counterparts.

## Introduction

Lung cancer is the second most diagnosed cancer worldwide. It’s the leading cause of cancer-related death in men while in women, it represents the second cause of death after breast cancer [1] with a cumulative 5-year survival of 10–20% [2].

Histologically it’s divided into two subtypes non-small cell lung cancer (NSCLC) which represents about 85% and small cell lung cancer (SCLC) which represent 15% [3]. NSCLC is further subdivided into non- squamous carcinoma (including adenocarcinoma, large-cell carcinoma, other cell types) and squamous cell carcinoma [4, 5].

Unfortunately NSCLC patients are mostly diagnosed at advanced stages (stage 3 and 4) which leads to poor prognosis [6].

Before the era of targeted and immunotherapy, platinum-based chemotherapy was the standard of care for NSCLC patients with advanced or metastatic disease. It consists of cisplatin or carboplatin in addition to taxanes, gemcitabine, vinorelbine, etoposide or pemetrexed [7, 8].

Cisplatin and carboplatin are heavy metal complexes that damage DNA molecules by crosslinking with the purine bases on the DNA; producing adducts that eventually cause apoptosis [9]. Interestingly, patients with the same histological types of tumor and of the same stage respond differently to chemotherapy, suggesting that there might be other factors contribute to chemoresistance such as genetic variants of certain genes involved in the response to chemotherapy [10].

Different pathways exist to repair DNA damage induced by platinum agents such as base excision repair pathway (BER), double strand break repair (DSB), DNA mismatch repair, and most importantly nucleotide excision repair (NER) [8, 10, 11]. Consequently single nucleotide polymorphisms (SNPs) in DNA repair genes may play an important role in response to chemotherapy, chemotherapy toxicity as well as cancer survival [6, 8, 10, 12].

Nucleotide excision repair (NER) enzymes are one of the main pathways involved in the repair process of platinum DNA adducts. It involves coordination of many enzymes that restore the platinum-induced DNA damage as excision repair cross-complementing group 1 (ERCC1), excision repair cross-complementation group 2 (ERCC2), excision repair cross-complementation group 5 (ERCC5) [8, 11].

Excision Repair Complementary Complex 5 (ERCC5), also known as the xeroderma pigmentosum complementation group G (XPG) gene, located on chromosome 13, is an important component of NER [13]. It contains 15 exons which forms a structure specific endonuclease that recognizes and cleaves the platinum—DNA adduct at both the 3′ and 5′ ends [14, 15]. Hence, change in the activity or expression of ERCC5 might affect the platinum activity which in turn affect response to platinum chemotherapy.

Most of the published studies have focused on the relation between single nucleotide polymorphisms (SNPs) in ERCC5 and the risk of various types of cancer [14–18] as well as cancer prognosis [19–21] while few studies have addressed the association between XPG polymorphisms and chemotherapy outcome [22, 23] and most of them were done on Asian populations.

Thus the objective of this study was to evaluate the effect of polymorphism in the ERCC5/XPG (rs751402 and rs1047768) gene on the clinical outcome of platinum-based regimens used in the treatment of NSCLC in Egyptian patients.

## Materials and methods

### Design and settings

A prospective, cohort study was conducted on 57 histologically confirmed non-small cell lung cancer (NSCLC) Egyptian patients recruited from the department of clinical oncology, faculty of medicine, Ain Shams University. Patient recruitment started from March 2017 to January 2019.

### Patients

Newly diagnosed histologically confirmed NSCLC patients receiving platinum-based chemotherapy with a performance status (PS) of 0–2 [24] and adequate bone marrow reserve were included in the study. Patients were excluded if they received previous chemotherapy or targeted therapy, had central nervous system metastases, inadequate liver, or kidney function, second primary malignancy, or were pregnant or lactating.

The study was approved by the Faculty of Pharmacy, Ain Shams University, Cairo, Egypt research ethics committee for experimental and clinical studies under number PHCL137. All the patients were required to give an informed consent for participation in the study without any obligations to withdraw from the study anytime. The study was conducted in accordance with the declaration of Helsinki as revised in 2013. The study was registered at clinical trial registry with Clinical trial.gov registration number: NCT03154242.

### Methods

#### Treatment and clinical assessments

Patients received intravenous doses of gemcitabine 1200 mg/m^2^ on day 1 and 8 or etoposide 100 mg/m^2^ day on day 1,2 and 3 plus cisplatin 75 mg/m^2^ or gemcitabine 1000 mg/m^2^ on day 1 and 8 or Paclitaxel 175 mg/m^2^ plus carboplatin AUC 5 on day 1 every 21 days per cycle for a maximum of 6 cycles. Chemotherapy was given only if neutrophils ≥ 1.5 × 10^9^/L, platelets ≥ 100 × 10^9^/L, and haemoglobin ≥ 9.0 gm/dL. Patients with poor performance or documented renal impairment or hearing loss were started with carboplatin rather than cisplatin-based regimen. Patients were shifted to carboplatin-based regimen during the chemotherapy cycles if they developed toxicities or could not tolerate it during subsequent cycles. Patients who experienced grade 3 or 4 toxicity had a change in therapy either dose reduction, discontinuation, or pharmacologic intervention to prevent or treat the toxicity according to the regimen protocol.

At baseline, patients’ demographic and clinical data were assessed including complete history, physical examination, radiographic investigations as CT scan (chest and pelvi-abdomen). Moreover, laboratory analysis including complete blood count (CBC) and liver and kidney function tests were performed. Patient prognostic stage was assessed using TNM 7th edition [25]. All laboratory analyses were repeated every cycle and CBC was repeated before day 8 of chemotherapy. Tumor response was evaluated using the Response Evaluation Criteria in Solid Tumors (RECIST) Version 1.1 [26].

The primary outcome was the progression-free survival (PFS); defined as the time from start of chemotherapy to the day of documented disease progression or death. While the secondary outcomes were objective response rate (ORR), and overall survival (OS); defined as the time from start of chemotherapy to death from any cause. Each patient was followed up to 18 months. Patients who completed the follow-up period without disease progression or death were considered censored. Patients who did not complete the follow-up period were censored at the last follow-up visit.

#### Polymorphism selection

Data about the allelic frequencies of different genes in the Egyptian population is still lacking, and hence we hypothesized its similarity to the European and African population distribution based on its location with respect to Africa and Europe. The single nucleotide polymorphisms (SNPs) were selected according to an average minor allele frequency (MAF) of more than or equal to 25% with higher frequencies in European or African population.

#### Sample collection and genotyping

Two millilitres of venous blood were withdrawn from each patient into vacutainer tubes containing Ethylene diamine tetra acetic acid (EDTA). DNA was isolated with the QIAamp DNA blood Mini kit (QIAGEN**®**, Germany), according to the manufacturer’s instructions [27]. For Single nucleotide polymorphism (rs1047768 and rs751402) genotyping; Taqman® Single nucleotide polymorphism (SNP) genotyping assay (Applied Biosystems®) [28] and TaqMan Universal PCR Master Mix (Applied Biosystems®) was used. Real-time PCR using Rotor gene Q (QIAGEN®) was performed.

#### Statistical analysis

Statistical analysis was done by the statistical software package version 20 (SPSS Inc., Chicago, IL). Frequencies and percentages were used to summarize categorical data. For assessment of agreement of genotype frequencies with those expected under Hardy–Weinberg equilibrium standard chi-square test was performed. Each polymorphism was analysed using general genetic model (homozygous major allele versus heterozygous versus homozygous minor allele) and those who were found statistically significant were further analysed using the dominant model (homozygous major allele compared to others) and recessive model (homozygous minor allele compared to others).

Chi-square and fisher’s exact tests were used to test if there was an association between the investigated polymorphisms and different clinicopathological variables or ORR. OS and PFS were evaluated with the Kaplan–Meier method and groups were compared with the log-rank test. All reported values were 2-sided and *p*-values < 0.05 were considered significant.

## Results

### Baseline characteristics

Patients’ characteristics at baseline are shown in Table 1. The median age of patients was 55 years (range 33–80), males comprised 80.7% of patients and 9 patients had 1st degree family history of cancer. Non-smokers represented 22.8% of patients. The patients’ tumor histologic subtypes were as follows; 47.4% adenocarcinoma, 36.8% squamous, and 15.8% other subtypes as large-cell or not otherwise specified. On presentation, 47 patients (82.5%) had advanced stage (stage 3b and 4) while 10 patients (17.5%) had earlier stages for which 3 of them had undergone surgery. Four patients of the study cohort undergone radical radiotherapy.

**Table 1:** Patient demographics, clinical and genotypic characteristics

Characteristics	Category	*n* (%)
Gender	Female	11 (19.3)
	Male	46 (80.7)
Median age (range)	55 (33–80)	
	< 55	26 (45.6)
	≥ 55	31 (54.4)
Comorbidities	Hypertension	10 (17.5)
	Diabetes	6 (10.5)
Smoking	Ever smoker	44 (77.2)
	Non-smoker	13 (22.8)
Family history (first degree)	No	48 (84.2)
	Yes	9 (15.8)
NSCLC subtype	Adenocarcinoma	27 (47.4)
	Squamous cell carcinoma	21 (36.8)
	Others	9 (15.8)
Performance status (PS)^a^	0	3 (5.3)
	1	33 (57.9)
	2	21 (36.8)
Stage^b^	Early (≤ 3A)	10 (17.5)
	Late (3B and 4)	47 (82.5)
Other treatment modalities	Surgery	3 (5.3)
	Radical radiotherapy	4 (7)
Chemotherapy regimen^c^	Platinum/Gemcitabine	51 (89.5)
	Platinum/Etoposide	5 (8.8)
	Platinum/Paclitaxel	1 (1.8)
Type of platinum	Cisplatin-based	39 (68.4)
	Carboplatin-based	18 (31.6)
Number of cycles	< 4	17 (29.8)
	≥ 4	40 (70.2)
rs751402	AA	5 (8.8)
	AG	25 (43.9)
	GG	27 (47.4)
rs1047768	CC	15 (26.3)
	CT	32 (56.1)
	TT	10 (17.5)

^a^Performance Status (PS) was assessed using Eastern Cooperative Oncology Group performance status (ECOG)

^b^Stage was assessed using AJCC TNM 7th edition

^c^Choice was based mainly on the availability in the hospital

At the start of chemotherapy, Performance status (PS) included 57.9% of patients PS 1 and 36.8% PS 2. Most of the platinum-based chemotherapy (68.4%) was cisplatin-based and 89.5% of patients received gemcitabine as their second agent. Most of the patients completed 4 or more cycles of chemotherapy.

### Genotype distribution and association between clinicopathologic variables and polymorphisms

Genotype frequencies of both ERCC5 rs751402 A > G and rs1047768 T > C are listed in Table 1 with minor alleles; A allele for rs751402 and T allele for rs1047768. Both frequencies are in concordance with those expected under Hardy–Weinberg equilibrium (*p* = 0.937 and *p* = 0.426, respectively).

On analysing the coinheritance between the genetic variants of the two variants as shown in Table 2, a statistically significant difference was found between them in every genetic model assessed (*p* = 0.00004, 0.000033, and 0.033 for general, dominant, and recessive models, respectively).

**Table 2:** Analysis of the coinheritance between the genetic variants of ERCC5

SNP	rs1047768			*p* value
	CC	CT	TT	
rs751402				
AA	0 (0)	2 (3.5)	3 (5.3)	**0.000040**^a^
AG	1 (1.8)	18 (31.6)	6 (10.5)	**0.000033**^b^
GG	14 (24.6)	12 (21.1)	1 (1.8)	**0.033**^c^

^a^*p* value was computed by fisher’s exact test comparing rs751402 variants to rs1047768 variants, bold indicate statistical significance

^b^*p* value was computed by chi-square test comparing rs751402 in dominant model (GG vs AA + AG) and rs1047768 in dominant model (CC vs TT + CT), bold indicate statistical significance

^c^*p* value was computed by fisher’s exact test comparing rs751402 in recessive model (AA vs GG + AG) and rs1047768 in recessive model (TT vs CC + CT), bold indicate statistical significance

Moreover, a statistically significant difference was evident between the various histologic subtypes and both rs751402 and rs1047768 genetic variants in the general model (*p* = 0.028 and 0.018, respectively). On further analysis, the association was found in rs751402 dominant genetic model; homozygous major allele GG compared to those with heterozygous and homozygous minor allele (AG + AA). However, this association was not evident in the recessive model (homozygous minor allele AA compared to the others (AG + GG) with *p* = 0.017. On the contrary, for rs1047768 variants the statistically significant difference was found in the recessive model between homozygous minor allele TT compared to (CC + CT) but not in the dominant model (CC vs TT + CT) with *p* = 0.014 as shown in Table 3

**Table 3:** Association between patients’ clinicopathological variables and genetic polymorphism of rs751402 and rs1047768 (*n* = 57)

Variable * n* (%)	rs751402 *n* (%)			*p* value	rs1047768 *n* (%)			*p* value
	AA	AG	GG		CC	CT	TT	
Gender								
Male	0 (0)	5 (8.8)	6 (10.5)	0.782^b^	1 (1.8)	7 (12.3)	3 (5.3)	0.376^b^
Female	5 (8.8)	20 (35.1)	21 (36.8)		14 (24.6)	25 (43.9)	7 (12.3)	
Age								
< 55	1 (1.8)	13 (22.8)	12 (21.1)	0.529^b^	7 (12.3)	15 (26.3)	4 (7)	0.926^a^
≥ 55	4 (7)	12 (21.1)	15 (26.3)		8 (10.4)	17 (29.8)	6 (10.5)	
Smoking								
Non-smoker	0 (0)	6 (10.5)	7 (12.3)	0.717^b^	2 (3.5)	7 (12.3)	4 (7)	0.346^b^
Ever smoker	5 (8.8)	19 (33.3)	20 (35.1)		13 (22.8)	25 (43.9)	6 (10.5)	
Family history								
No	4 (7)	23 (40.4)	21 (36.8)	0.337^b^	13 (22.8)	25 (43.9)	10 (17.5)	0.290^b^
Yes	1 (1.8)	2 (3.5)	6 (10.5)		2 (3.5)	7 (12.3)	0 (0)	
Subtype								
Adeno	3 (5.3)	12 (21.1)	12 (21.1)	**0.028**^**b**^	5 (8.8)	19 (33.3)	3 (5.3)	**0.018**^**b**^
Squamous	0 (0)	7 (12.3)	14 (24.6)	**0.017**^b*^	8 (14)	11 (19.3)	2 (3.5)	0.297^a*^
Other	2 (3.5)	6 (10.5)	1 (1.8)	0.098^b**^	2 (3.5)	2 (3.5)	5 (8.8)	**0.014**^b**^
ECOG PS								
0	0 (0)	2 (3.5)	1 (1.8)	0.853^b^	1 (1.8)	2 (3.5)	0 (0)	0.788^b^
1	4 (7)	14 (24.6)	15 (26.3)		7 (12.3)	20 (35.1)	6 (10.5)	
2	1 (1.8)	9 (15.8)	11 (19.3)		7 (12.3)	10 (17.5)	4 (7)	
Stage^†^								
Early (≤ 3A)	1 (1.8)	5 (8.8)	4 (7)	0.882^b^	3 (5.3)	5 (8.8)	2 (3.5)	0.901^b^
Late (3B & 4)	4 (7)	20 (35.1)	23 (40.4)		12 (21.1)	27 (47.4)	8 (14)	
Type of platinum								
Cisplatin	5 (8.8)	19 (33.3)	15 (26.3)	0.096^b^	10 (17.5)	23 (40.4)	6 (10.5)	0.797^b^
Carboplatin	0 (0)	6 (10.5)	12 (21.1)		5 (8.8)	9 (15.8)	4 (7)	
No. of cycles								
< 4	2 (3.5)	7 (12.3)	8 (14)	0.833^b^	3 (5.3)	10 (17.5)	4 (7)	0.487^b^
≥ 4	3 (5.3)	18 (31.6)	19 (33.3)		12 (21.1)	22 (38.6)	6 (10.5)	

*ECOG PS* Eastern Cooperative Oncology Group performance status

*p value obtained from analysis of dominant model (GG vs AA + AG) for rs751402 and (CC vs TT + CT) for rs1047768

**p value obtained from analysis of recessive model (AA vs GG + AG) for rs751402 and (TT vs CC + CT) for rs1047768

^**†**^Stage was assessed using AJCC TNM 7th edition

^a^Chi-square test, two-sided *p* value < 0.05 is significant, ^b^ Fisher's exact test, two-sided *p* value < 0.05 is significant, bold indicates statistical significant

### Response analysis

Among the studied patients, none achieved complete response (CR) while 6 patients (10.5%) had partial response (PR), 18 patients (31.6%) had stable disease (SD) and 23 patients (40.4%) had progressed disease (PD). Nine patients (15.8%) had not undergone response assessment; 3 patients (5.3%) of them due to receiving chemotherapy as neoadjuvant or adjuvant and 6 patients (10.5%) due to patient’s death or loss to follow-up before undergoing first CT scan during chemotherapy cycles.

No statistically significant difference was observed between genetic polymorphisms of ERCC5 rs751402 and rs1047768 and patients’ response to chemotherapy as shown in Table 4

**Table 4:** Response analysis of rs751402 and rs1047768 polymorphisms (*n* = 57)

Response	Tumor response^a,b^ *n* (%)			*p* value^c^	Response category^a, b^ *n* (%)		*p* value^c^
	PR	SD	PD		Responder (CR + PR)	Non-responder (SD + PD)	
rs751402				0.369			0.217
AA	1 (1.8)	1 (1.8)	3 (5.3)		1 (1.8)	4 (7)	
AG	4 (7)	6 (10.5)	10 (17.5)		4 (7)	16 (28)	
GG	1 (1.8)	11 (19.3)	11 (19.3)		1 (1.8)	22 (38.6)	
rs1047768				0.574			1.00
CC	2 (3.5)	7 (12.3)	4 (7)		2 (3.5)	11 (19.3)	
CT	3 (5.3)	8 (14)	15 (26.3)		3 (5.3)	23 (40.4)	
TT	1 (1.8)	3 (5.3)	5 (8.8)		1 (1.8)	8 (14)	

*CR* complete response, *PD* progressed disease, *PR* partial response, *SD* stable disease

^a^Nine (15.8%) patients have not undergone response assessment

^b^Response was assessed using RECIST 1.1

^c^Fisher's exact test, two-sided *p* value < 0.05 is significant

### Survival analysis

*Progression-free survival* (*PFS*) at the end of the 18 months period, 43 patients (75.4%) had progressed, 3 patients had not shown any disease progression and 11 patients (19.3%) were lost to follow-up while for *Overall survival* (*OS*) 20 patients (35.1%) died, 22 patients (38.6%) completed the follow-up period, while 15 patients (26.3%) were lost to follow-up.

**Fig. 1:**
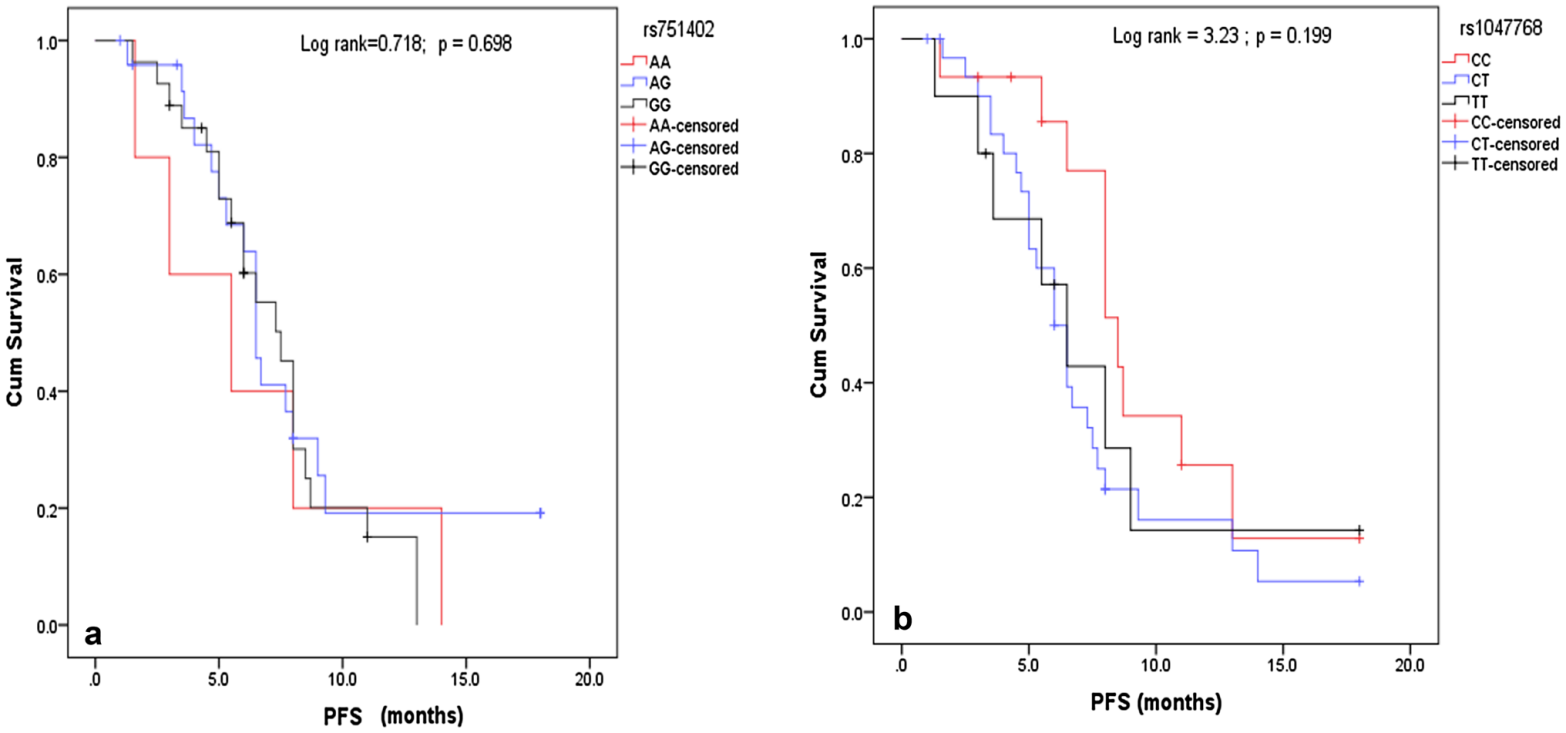
Kaplan Meier curve showing the effect of genetic variants on Progression-Free Survival (PFS). **a** Curve represents the relation between rs751402 variants (AA vs AG vs GG) and PFS. **b** Curve represents the relation between rs1047768 variants (CC vs CT vs TT) and PFS

**Fig. 2:**
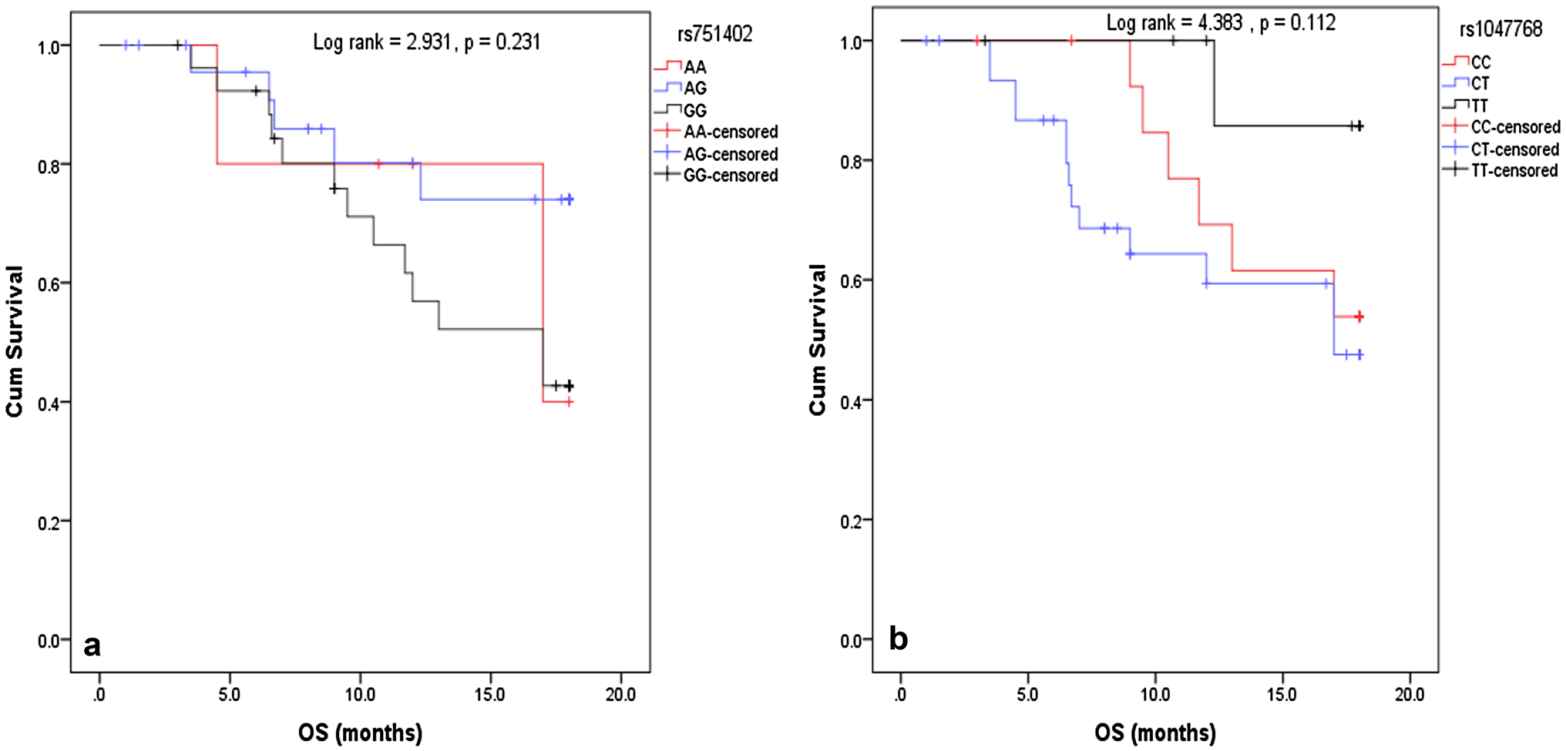
Kaplan Meier curve showing the effect on Overall Survival (OS). **a** Curve represents the relation between rs751402 variants (AA vs AG vs GG) and OS. **b** Curve represents the relation between rs1047768 variants (CC vs CT vs TT) and OS

There was not any statistically significant difference between PFS or OS and each of rs751402 and rs1047768 polymorphisms as shown in Figs. 1 and 2

## Discussion

Non-small cell lung cancer (NSCLC) is one of the leading causes of cancer-related deaths [1, 4]. Platinum-based chemotherapy is essential for treatment of NSCLC patients without driver mutations and for those who cannot afford the cost of targeted and immunotherapy [29]. Since the response to platinum-based regimens is variable therefore the need for identifying patients who would benefit more from chemotherapy is crucial.

Enzyme repair complementary complex 5 (ERCC5) is one of the key players for repairing platinum DNA adduct through the dual incision at both the 5’ and 3’ sides of the adduct which may lead to a decrease in platinum activity against cancer [30]. Hence, mutations in ERCC5 might enhance platinum effect on cancer cells. The current study is one of the few studies that addressed the relation between ERCC5 polymorphisms in rs751402 and rs1047768 and the outcome of platinum-based chemotherapy in terms of response and survival. Moreover, most of the published studies have focused on Asian populations rather than Caucasians.

The polymorphisms of rs751402 and rs1047768 both followed the Hardy–Weinberg distribution with a minor allele frequency (MAF) of 29.7% for the A allele in rs751402 which resembles the MAF of this allele in the African population (30–31%). While the T allele, which is the minor allele of rs1047768, showed a frequency of 41.8% which resembles the MAF of the same allele in the European population (41.2%) according to the alfa project from NCBI [31, 32]. Data regarding the allelic frequencies and ethnicity origin of the Egyptian population is still lacking, with a proposed heterogenous distribution from African–European ancestry. Hence, the results of the current study could pave the way when interpreting the scarce data addressing the Egyptian population. Moreover, it can guide future research when extrapolating data from African European populations to their Egyptian counterparts.

On investigating the coinheritance between the studied polymorphisms, we found a statistically significant association in the general genetic model as well as the dominant and recessive models as shown in Table 2. These associations might be attributed to the effect of rs751402, located in the promotor region, on the rs1047768, located in the exon region, indicating that rs751402 variants might affect the type of the allele present on rs1047768. Similarly, Blomquist et al. in their study on normal bronchial epithelial cells found that the A allele on rs751402 was associated with higher expression of T allele on rs1047768 [33].

On studying the association between the clinicopathologic variables and rs751402 or rs1047768 genetic variants, no statistically significant difference was found apart from the histologic subtypes. Interestingly, there was a statistically significant association between the histologic subtypes and the studied genotypes in some of the genetic models. Regarding rs751402, a relation between the histologic subtypes and different genotypes was found in the general model and dominant model. This might indicate that the A allele (minor allele) was more expressed with specific histologic subtypes. On the other hand, for the rs1047768, the association was found in the general and recessive model representing a link with the C allele (major allele). Rulli et al. found a similar association between ERCC1 (rs11615) genetic variants and the histologic subtypes of NSCLC [34].

The current study did not report an association between any of the investigated SNPs and the platinum response. Similarly, other NSCLC patients of different ethnicities did not report an association [35, 36]. However, a metanalysis in NSCLC found that patients with C allele in rs1047768 were more platinum sensitive [37]. Similarly, He et al. in their study on Chinese population with NSCLC found that AA genotype of rs751402 had better platinum response than AG + GG genotypes specially in the squamous subtype [38]. The discrepancies between those studies and ours might be attributed to the racial differences between the two populations.

In accordance with what was found in other studies [22, 34], the current study showed no statistically significant difference between the different genotypes in both SNPs and neither PFS nor OS. On the contrary, these findings contradict what was found in a trial on NSCLC patients where the TT genotype of rs1047768 had statistically significant shorter PFS and OS compared to the CC genotype [39]. This difference in relation to our findings might be attributed to several factors including greater sample size and differences in the genetic makeup between the populations.

## Conclusion and limitation

This study couldn’t find an association between the polymorphisms of ERCC5 rs751402 or rs1047768 and the outcome of platinum-based chemotherapy, however, it showed an association between rs751402 and rs1047768 genetic variants and the histologic subtypes of NSCLC. Moreover, a strong association was evident between the type of the allele present in the two selected SNPs. Precision medicine aims at targeting therapy, improving outcomes & subsequently reducing costs. Hence, identifying those variants that will affect and those that will not affect are both important in guiding therapy decisions. The coinheritance among the studied genetic variants might suggest a linkage disequilibrium among them which might pave the way for further research of finding tag SNPs that could predict response. The similarity between the MAF of the studied variants and the African or European population might shed the light on further understanding of the ethnicity of the Egyptian population. This study is limited by the small sample size and short follow-up period. Hence, larger studies with longer follow-up is needed to confirm the results of this study and to better elucidate the relationship between the two studied variants.
